# Soluble PD-1 but Not PD-L1 Levels Predict Poor Outcome in Patients with High-Risk Diffuse Large B-Cell Lymphoma

**DOI:** 10.3390/cancers13030398

**Published:** 2021-01-22

**Authors:** Heli Vajavaara, Julie Bondgaard Mortensen, Suvi-Katri Leivonen, Ida Monrad Hansen, Maja Ludvigsen, Harald Holte, Judit Jørgensen, Mette Bjerre, Francesco d’Amore, Sirpa Leppä

**Affiliations:** 1 Research Program Unit, Applied Tumor Genomics Research Program, Faculty of Medicine, University of Helsinki, 00014 Helsinki, Finland; heli.vajavaara@helsinki.fi (H.V.); suvi-katri.leivonen@helsinki.fi (S.-K.L.); 2 Department of Oncology, Helsinki University Hospital Comprehensive Cancer Center, 00029 Helsinki, Finland; 3 iCAN Digital Precision Cancer Medicine Flagship, 00014 Helsinki, Finland; 4 Department of Hematology, Aarhus University Hospital, Aarhus, DK-8200 Aarhus N, Denmark; jumort@rm.dk (J.B.M.); idprha@rm.dk (I.M.H.); majlud@rm.dk (M.L.); Judit.Joergensen@aarhus.rm.dk (J.J.); frandamo@rm.dk (F.d.); 5 Department of Clinical Medicine, Aarhus University Hospital, Aarhus, DK-8200 Aarhus N, Denmark; 6 Department of Oncology and KG Jebsen Center for B-Cell Malignancies, Oslo University Hospital, 0424 Oslo, Norway; hhe@ous-hf.no; 7 Medical Research Laboratory, Aarhus University, DK-8200 Aarhus N, Denmark; mette.bjerre@clin.au.dk

**Keywords:** soluble PD-1, soluble PD-L1, diffuse large B-cell lymphoma, survival

## Abstract

**Simple Summary:**

Soluble forms of checkpoint protein PD-1 and its ligand PD-L1 can be measured from circulation, but their source, function, and clinical impact in cancer remain incompletely understood. In this study, we used serum samples collected during a conduction of a prospective immunochemotherapy trial in patients with high-risk diffuse large B-cell lymphoma (DLBCL) and assessed their clinical significance. Our results demonstrate that sPD-1 levels in the peripheral blood at the time of diagnosis correlate with the quantities of tumor infiltrating PD1+ T cells and translate to inferior survival. To our knowledge, this is the first study to identify sPD-1 as a prognostic factor, providing interesting perspectives on future clinical trials in DLBCL, including patients’ stratification associated with checkpoint blockade.

**Abstract:**

Interaction of checkpoint receptor programmed death 1 (PD-1) with its ligand 1 (PD-L1) downregulates T cell effector functions and thereby leads to tumor immune escape. Here, we aimed to determine the clinical significance of soluble PD-1 (sPD-1) and soluble PD-L1 (sPD-L1) in patients with diffuse large B-cell lymphoma (DLBCL). We included 121 high-risk DLBCL patients treated in the Nordic NLG-LBC-05 trial with dose-dense immunochemotherapy. sPD-1 and sPD-L1 levels were measured from serum samples collected prior to treatment, after three immunochemotherapy courses, and at the end of therapy. sPD-1 and sPD-L1 levels were the highest in pretreatment samples, declining after three courses, and remaining low post-treatment. Pretreatment sPD-1 levels correlated with the quantities of PD1+ T cells in tumor tissue and translated to inferior survival, while no correlation was observed between sPD-L1 levels and outcome. The relative risk of death was 2.9-fold (95% CI 1.12–7.75, *p* = 0.028) and the risk of progression was 2.8-fold (95% CI 1.16–6.56, *p* = 0.021) in patients with high pretreatment sPD-1 levels compared to those with low levels. In conclusion, pretreatment sPD-1 level is a predictor of poor outcome after dose-dense immunochemotherapy and may be helpful in further improving molecular risk profiles in DLBCL.

## 1. Introduction

Approximately two-thirds of patients with diffuse large B-cell lymphoma (DLBCL) are cured in response to standard rituximab, cyclophosphamide, doxorubicin, vincristine, and prednisone (R-CHOP)-based immunochemotherapy [1]. However, treatment options for primary refractory and relapsed patients are few, and the disease is often fatal. If molecular factors determining the poor prognosis were understood better, targeted therapies could be applied not only to improve outcomes in these patients but also to avoid overtreatment.

DLBCL is a heterogeneous disease. Recent comprehensive genome and transcriptome studies elucidated the heterogeneity of DLBCL and revealed multiple DLBCL subtypes with different outcomes [2,3,4]. In addition to tumor-cell-derived factors, an association between tumor microenvironment (TME) and survival was identified. Among the most essential prognostic microenvironmental factors are tumor-infiltrating T cells (TILs) [5,6,7].

Antitumor T cell responses are critical for immune surveillance of malignant diseases, but they may be counterbalanced by a number of immune checkpoints used by tumors to actively evade immune destruction [8]. Among all immune checkpoints, the programmed cell death 1 (PD-1; also called CD279)/programmed death ligand 1 (PD-L1; CD274) pathway stands out because of its value as a therapeutic target in many malignancies [9,10]. PD-1 interacts with PD-L1 and PD-L2 (CD273), of which PD-L1 is considered as a dominant inhibitory ligand of PD-1 in human TME. While PD-1 is expressed mainly on activated T cells, PD-L1 expression is broader, including tumor cells, tumor infiltrating immune cells, and cells in circulation, such as neutrophils [11]. Interaction of PD-L1 with PD-1 results in downregulation of T cell effector functions and inhibition of the antitumor immune response [10,11,12].

In DLBCL, roles of PD-1 and PD-L1 expression remain obscure, as, depending on assessment method, target cells (TME or tumor cells), and used cutoff levels, some studies found high amounts of PD-1+ TILs or PD-L1 expression to be associated with poor outcomes, whereas other studies showed favorable or no prognostic impact on survival [12,13,14,15,16,17,18,19].

In addition to full-length, membrane-bound isoforms, alternatively spliced PD-1 and PD-L1 transcripts were described [20,21,22]. A splice variant PD-1△ex3 lacking the transmembrane domain results into production of soluble PD-1 (sPD-1). sPD-1 bind to PD-L1/L2 in vitro, thereby blocking interaction between membrane-bound PD-1 (mPD-1) and PD-L1/L2 [23]. This could lead to inhibition of negative signals provided by mPD-1 and expose cancer cells to immune surveillance. Consistent with the hypothesis that sPD-1 could act as a checkpoint inhibitor was the finding that coadministration of sPD-1-encoding DNA with two different vaccines enhanced antigen-specific CD8+ T cell responses, resulting in antitumor effects in mice [24]. Furthermore, sPD-1 expressing senescent tumor cell vaccine delayed tumorigenesis and suppressed tumor growth in a triple-negative breast cancer mouse model [25]. Similarly, a splice variant CD274-L2A resulted in a membrane-free, soluble form of PD-L1 (sPD-L1) [21,22]. sPD-L1 might also result from cleavage of membrane-bound PD-L1 (mPD-L1) [26]. Recently, it was shown that although sPD-L1 retains PD-1 binding activity on T cells, it lacks measurable T cell inhibitory activity. Instead, sPD-L1 can act as a PD-1 antagonist by reversing T cell suppression mediated by mPD-L1 [27].

sPD-1 and sPD-L1 can be measured from different body fluids, including serum [28,29,30,31,32], but their source, function, and clinical impact in cancer remain incompletely understood. In patients with resected hepatocellular carcinoma, elevated sPD-1 levels were shown to be associated with favorable outcomes [29]. High serum sPD-L1 levels were in turn associated with poor outcomes in patients with DLBCL [19,31,33]. We previously observed elevated pretreatment sPD-1 levels in DLBCL patients compared to healthy controls [34]. In this study, we used serum samples collected during conduction of a prospective DLBCL trial at three different time points, allowing us to analyze sPD-1 and sPD-L1 levels during immunochemotherapy and assess whether the levels were associated with outcome.

## 2. Results

### 2.1. Clinical Characteristics

The Nordic Lymphoma Group Large B-Cell (NLG-LBC-)05 trial included 139 patients aged 18–64 years, who had primary DLBCL with age-adjusted International Prognostic Index (aaIPI) 2–3 or site-specific risk factors for central nervous system (CNS) recurrence [35]. The patients were treated with dose-dense immunochemotherapy and systemic early CNS prophylaxis [35]. Patient demographics of the study cohort (*n* = 121) were representative of the entire clinical trial (Table 1). Most of the patients had DLBCL not otherwise specified (NOS, *n* = 105, 87%), eight (7%) patients had primary mediastinal B-cell lymphoma, three (2%) had grade IIIb follicular lymphoma, four (3%) had T cell/histiocyte-rich large B-cell lymphoma, and one (1%) had intravascular large B-cell lymphoma. No EBV+ DLBCL NOS cases were included. The median age was 56 years (range 21–65 years). The majority of the patients were males with good Eastern Cooperative Oncology Group (ECOG) performance scores and advanced stage disease. In addition, 74 (61%) patients had B symptoms and 110 (91%) had elevated lactate dehydrogenase (LDH). During the median follow up time of 61 months (range 40–85 months), 18 (15%) patients relapsed and 16 (13%) died, translating to 87% OS and 83% PFS rates at five years. Twelve (10%) of the deaths were lymphoma-related.

### 2.2. sPD-1 and sPD-L1 Levels During Therapy

**Figure 1:**
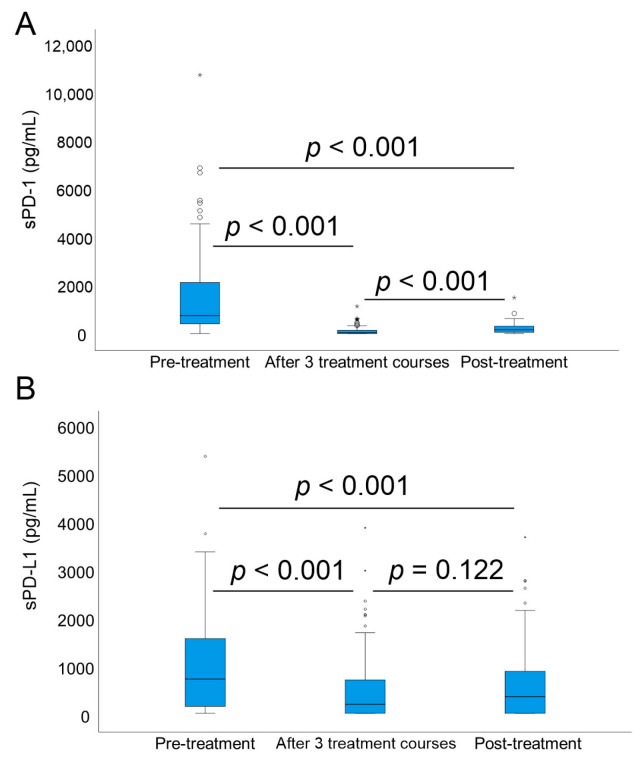
sPD-1 and sPD-L1 levels during different time points. Pretreatment, after three treatment courses, and post-treatment levels of (**A**) soluble PD-1 and (**B**) soluble PD-L1 and the significance of the level change between these three time points *.

sPD-1 and sPD-L1 levels were measured at three different time points. The highest sPD-1 levels were observed pretherapeutically (median 973 pg/mL, range 40 pg/mL to 10,761 pg/mL). A significant decline in sPD-1 levels compared to pretherapeutic levels was observed in response to immunochemotherapy after three treatment courses (median 81 pg/mL, range 40–1163 pg/mL, *p* < 0.001) and after all courses (median 185 pg/mL, range 40–1525 pg/mL, *p* < 0.001; Figure 1A). Post-treatment levels were higher than levels during therapy (*p* < 0.001; Figure 1A). Similarly, sPD-L1 levels were highest pretherapeutically (median 766 pg/mL, range 56–5393 pg/mL), declining during therapy (median 244 pg/mL, range 56–3905 pg/mL, *p* < 0.001), and remaining significantly lower after all courses (median 402 pg/mL, range 56–3712 pg/mL; Figure 1B) compared to pretreatment levels (*p* < 0.001). No correlation was seen between sPD1 and sPD-L1 levels (in all comparisons, *p* > 0.43).

### 2.3. Correlation of sPD-1 and sPD-L1 Levels with Clinical Parameters, Gene and Protein Expression and Lymphocyte Count

**Figure 2:**
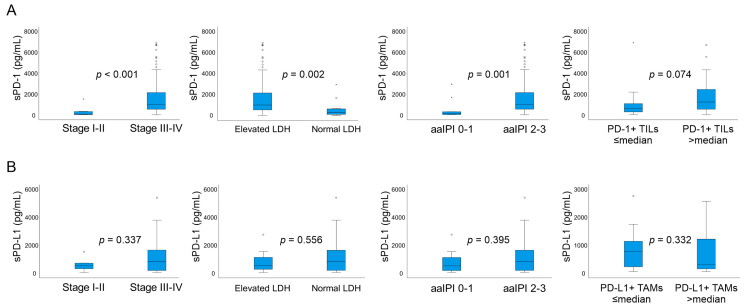
Pretreatment sPD-1 and sPD-L1 levels according to different parameters. (**A**) Correlation between pretreatment sPD-1 levels and clinical stage, lactate dehydrogenase (LDH) level, age-adjusted International Prognostic Index (aaIPI) score, and PD-1+ tumor infiltrating T lymphocytes (TILs). (**B**) Correlation between pretreatment sPD-L1 levels and clinical stage, LDH level, aaIPI score, and PD-L1+ tumor-associated macrophages (TAMs). sPD-1 and sPD-L1 values were analyzed as continuous variables and the levels between the groups were compared with the Mann–Whitney U test.

Patients with advanced stage (III-IV), elevated LDH, and high aaIPI (2–3) score had higher pretreatment sPD-1 levels compared to patients with low stage (I-II), normal LDH, and low aaIPI (0–1), respectively (Figure 2A). In addition, sPD-1 levels tended to be higher in patients who had high PD1+ T cell contents in their tumor tissue (>median, Figure 2A), whereas no differences were observed in pretreatment sPD-1 levels between good and poor performance scores (0–2 vs. 3–4), gender, age (<60 years vs. 60–65 years), or molecular subtype. In comparison, pretreatment sPD-L1 levels analyzed as continuous variables (Mann-Whitney U test, *p* = 0.002) were higher in patients with poor ECOG PS, whereas no correlation was seen between other clinical parameters or PD-L1+ TAM count and sPD-L1 levels (Figure 2B).

**Figure 3:**
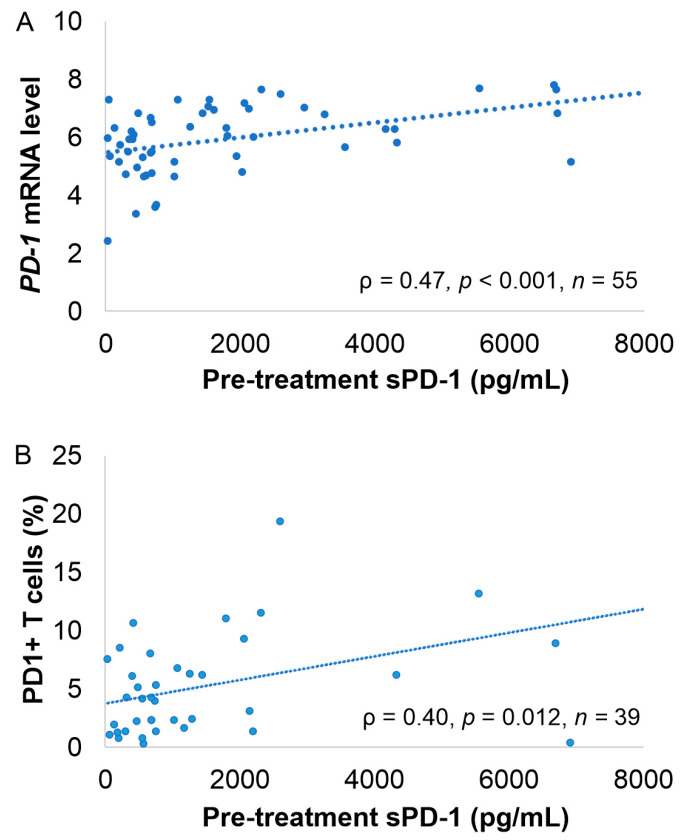
Correlation between pretreatment sPD-1 levels, PDCD1 gene expression levels, and PD1+ tumor-infiltrating T lymphocytes. (**A**) Correlation between pretreatment sPD-1 levels and *PDCD1* mRNA levels from the tumor tissue. (**B**) Correlation between pretreatment sPD-1 levels and proportion of PD1+ T cells in the tumor tissue. Correlation analyses were performed with Spearman rank analysis.

Pretreatment sPD-1 level correlated with *PDCD1* gene expression in matching tumor tissue (*ρ* = 0.467, *p* < 0.001; Figure 3A). In addition, we found a positive correlation between pretreatment sPD-1 levels and quantities of tumor infiltrating PD-1+ T cells (*ρ* = 0.396, *p* = 0.012, *n* = 39; Figure 3B), PD-1+ T-helper cells (*ρ* = 0.433, *p* = 0.019, *n* = 29), PD-1+ cytotoxic T cells (*ρ* = 0.385, *p* = 0.039, *n* = 29), and PD-L1+ tumor-associated macrophages (TAMs) (*ρ* = 0.397, *p* = 0.012, *n* = 39). In contrast, no correlation was found between pretreatment sPD-1 levels and PD-1+ TAMs (*p* = 0.6, *n* = 39), nor blood absolute lymphocyte counts (*p* = 0.6, *n* = 102). Neither did we observe any correlation between sPD-L1 levels and *PD-L1* gene-expression level (*p* = 0.80) or tumor infiltrating PD-L1+ cells (*p* = 0.92) or PD-L1+ TAMs (*p* = 0.57).

### 2.4. Association of sPD-1 and sPD-L1 Levels with Survival

**Figure 4:**
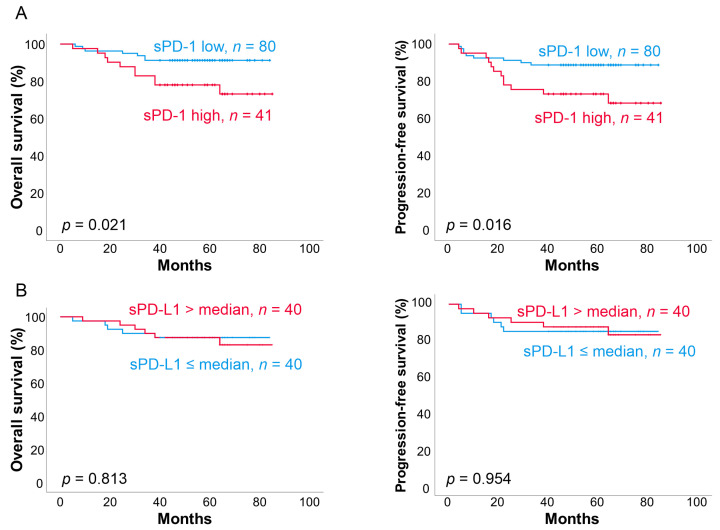
Survival according to pretreatment sPD-1 and PD-L1 levels. (**A**) Overall survival (OS) and progression-free survival according to low (<1565 pg/mL) and high (>1565 pg/mL) pretreatment sPD-1 levels. (**B**) OS and PFS according to low (≤median) and high (>median) pretreatment sPD-L1 levels.

According to a receiver operating characteristics (ROC) analysis, a pretreatment sPD-1 cutoff level of 1565 pg/mL, corresponding to 66%, best separated the two subgroups with different outcomes. Patients with high pretreatment sPD-1 levels (highest third, “high sPD-1 group”) had significantly worse outcomes than patients with low pretreatment sPD-1 levels (lowest two-thirds, “low sPD-1 group”) (five-year OS 78% vs. 91%, *p* = 0.021; five-year PFS 73% vs. 89%, *p* = 0.016; Figure 4).

In the high sPD-1 group, relative risk of death was increased 2.9-fold (95% CI 1.12–7.75, *p* = 0.028) and risk of progression by 2.8-fold (95% CI 1.16–6.56, *p* = 0.021), compared to the low sPD-1 group. Patients in the high sPD-1 group more often demonstrated advanced stage compared to the low sPD-1 group (*p* = 0.050). However, the prognostic impact of sPD-1 levels on OS (HR 2.64, 95% CI 1.01–6.94, *p* = 0.049) and PFS (HR 2.47, 95% CI 1.04–5.86, *p* = 0.040) was sustained when adjusted for stage. Other baseline characteristics were equally distributed between sPD-1 high and low subgroups (Table 1). In multivariate analysis with age, ECOG performance score, stage, LDH level, and molecular subtype, pretreatment sPD-1 level was retained as the only prognostic factor for OS and PFS (Table 2). In comparison, pretreatment sPD-L1 levels were not associated with survival in this cohort (Figure 4B). The patients with high pretreatment sPD-L1 levels (>median) had worse ECOG performance scores more often (PS 2–3: *p* = 0.005) and higher aaIPI scores (*p* = 0.016) than the patients with low pretreatment sPD-L1 levels. Otherwise, baseline characteristics were equally distributed between sPD-L1 high and low subgroups (Table 1). Neither the levels of sPD-1 nor sPD-L1 levels measured after three treatment courses or post-treatment translated to poor outcome.

## 3. Discussion

Although the role of mPD-1 as a key immune-checkpoint protein expressed on activated T cells is well established, and the PD-1△ex3 splice variant encoding sPD-1 was originally described 15 years ago [20], the function and clinical impact of sPD-1 remain unclear. Particularly in hematologic malignancies, data on sPD-1 are limited. Furthermore, while there are data showing correlation of sPD-L1 with outcome in patients with DLBCL [31], the clinical impact of sPD-L1 in the high-risk subgroup in combination with sPD-1 was not previously addressed. As the clinical course of the patients with DLBCL is heterogeneous, even within different International Prognostic Index risk groups, novel, easily measurable molecular tools for risk stratification would be needed. Measuring soluble biomarkers at diagnosis or during the course of therapy is practical, as blood samples can be collected easily from all patients.

This study was established along with a prospective clinical trial exploring whether dose-dense immunochemotherapy and early CNS prophylaxis improves outcomes and reduces the incidence of CNS events in patients with high-risk DLBCL [35]. We determined sPD-1 and sPD-L1 levels in serum samples before, during, and after therapy, and correlated the findings with baseline characteristics and survival. We observed that the levels of both sPD-1 and sPD-L1 were highest pretherapeutically, declining significantly in response to immunochemotherapy, and remaining low after therapy. The pretreatment sPD-1 levels were similar to the levels we detected previously in another DLBCL cohort [34]. The findings were also consistent with a small study on classical Hodgkin lymphoma, demonstrating a significant reduction in sPD-1 levels in response to therapy [30]. The reason for the decline in sPD-1 and sPD-L1 levels during the course of therapy is unknown; nevertheless, we speculate that it is due to the cytoreductive and immunosuppressive effect of the dose-dense treatment on T cells and other hematologic cell types, particularly neutrophils [21].

Opposite to our previous finding that PD-1 expression in the tumor tissue does not translate to adverse outcome [15], we observed that pretreatment sPD-1 levels correlated with more aggressive disease and unfavorable outcomes in our selected, clinically high-risk patient population. Nevertheless, high pretreatment sPD-1 level was the only prognostic factor for OS and PFS. Considering the correlation between pretreatment sPD-1 levels and quantity of tumor infiltrating PD-1+ T cells in our cohort, we speculate that increased sPD-1 levels can indicate systemic inflammation provoked by inflammatory tumor cells. In addition, it is possible that sPD-1 is derived at least partially from tumor-infiltrating T cells. However, the phenotype of these T cells remains to be characterized. It is also plausible that pretreatment sPD-1 levels mirror T cell exhaustion, thereby connecting to the detrimental clinical course of the disease. In contrast, we did not observe any correlation between sPD-L1 levels and tumor-derived PD-L1 expression, implying that sPD-L1 is not a simple surrogate of PD-L1 expression by the tumor tissue. Similar to our findings, Keane et al. observed no correlation between sPD-L1 and PDL1+ T cells in the tumor tissue [36]. In addition, apart from the patients with high sPD-L1 levels showing poor performance scores (ECOG 2–3) more often than the patients with low sPD-L1 levels, we could not find any association of sPD-L1 levels with clinical characteristics or outcome. This observation differs from a study where sPD-L1 levels were shown to correlate with unfavorable outcome in response to R-CHOP [31]. Our data are, however, similar to the finding from the validation cohort of the same study, where the patients were treated uniformly with high-dose chemotherapy supported with autologous transplantation [31]. These results indicate a possible attenuation of the high sPD-L1 effect on the prognosis by the high-dose intensity treatment.

Our results were opposite to the initial hypothesis based on the potential of sPD-1 to act as a checkpoint-inhibitor-like protein by binding to mPD-L1 and mPD-L2. Supporting our finding, natural sPD-1 was shown to have a low binding affinity to mPD-L1 and mPD-L2 [37], and therefore high-affinity PD-1 molecules were developed [38]. Thus, it seems likely that although sPD-1 levels are high prior to treatment, they do not translate to a similar outcome as checkpoint inhibitors due to weak sPD-1 binding affinity. Considering that PD-L1 is expressed on TAMs, apart from binding to PD-L1 on tumor cells, sPD-1 might also bind to PD-L1 on TAMs. Along with our observations, responses to a PD-1 antibody, nivolumab, were shown to be low in a recent phase II trial for the patients with relapsed/refractory DLBCL [39]. Conversely, PD-L1 gene alterations were associated with better response to pembrolizumab [17]. Thus, it was suggested that low PD-L1 expression on lymphoma cells would explain, at least to some extent, why the majority of the DLBCL patients do not benefit from checkpoint blockade [17,39,40]. This may also explain why sPD-1 does not have significant antitumor activity in DLBCL. Given that sPD-L1 can be detected in peripheral blood, it is also possible that the interaction between sPD-1 and sPD-L1 blocks the interaction between sPD-1 and mPD-L1. Taken together, it seems likely that, apart from predicting outcome, sPD-1 and sPD-L1 and possibly their ratio represent another unanticipated element contributing to final immune response.

Our study demonstrates that high sPD-1 levels in peripheral blood at the time of diagnosis are associated with poor survival in patients diagnosed with clinically high-risk DLBCL treated uniformly in a multicenter clinical trial. Our findings should be confirmed in a large independent cohort. Nevertheless, to our knowledge, this is the first study to identify sPD-1 as a prognostic factor, providing interesting perspectives on future clinical trials in DLBCL, including patients’ stratification associated with checkpoint blockade.

## 4. Materials and Methods

### 4.1. Patients

The study population consisted of 121 young, high-risk patients with primary DLBCL from a Nordic multicenter phase II NLG-LBC-05 trial [35] selected based on the availability of serum samples. The patients were treated with biweekly rituximab, cyclophosphamide, doxorubicin, etoposide, and prednisone (R-CHOEP) immunochemotherapy and systemic early central nervous system (CNS) prophylaxis (high-dose (HD) methotrexate (Mtx) and HD-cytarabine) [35]. Pretreatment serum samples for sPD-1 measurement were available from all patients (*n* = 121), after three treatment courses from 95 patients and after all courses from 98 patients, and for sPD-L1 measurement from 80 patients at all three time points. All patients signed informed consent before study participation. The Institutional Review Boards, National Medical Agencies, and Ethics Committees in Finland, Norway, Denmark, and Sweden approved the protocol and sampling. The trial was registered at www.ClinicalTrials.gov, number NCT01325194.

### 4.2. Measurement of sPD-1 and sPD-L1 Levels

sPD-1 levels were measured from the serum samples using a self-established Time-Resolved Immunofluorometric Assay (TRIFMA) based on commercial antibodies. Wells of 96-well plates were coated with antihuman PD-1 antibodies (1 µg/mL) in phosphate-buffered saline (PBS) (pH 7.4), and residual binding sites were blocked for 1 h with PBS, 1% Tween20 (Tw). Recombinant PD-1 in the range from 39 to 2500 pg/mL was used as a standard (Bio-Techne #DY1086, R&D Systems, Abingdon, UK). Serum samples were diluted 4-fold in the assay buffer (PBS with 5% skim milk, 1% bovine serum albumin, 2% normal goat serum and 0.05% Tw). Bound PD-1 was determined by incubation with biotin-labeled antihuman PD-1 antibody (200 ng/mL) in the assay buffer, followed by addition of 10 ng Eu3+-labeled streptavidin (Perkin Elmer Life Sciences, Turku, Finland). Bound europium was detected by the addition of 200 µL of enhancement solution (Perkin Elmer Life Sciences, Turku, Finland) and reading the time-resolved fluorescence on a DELFIA fluorometer (Victor, Perkin Elmer, MA, USA). All samples were analyzed in duplicate. The detection limit was 40 pg/mL, as previously reported, and samples below this cutoff were assigned this value [41]. Wells receiving buffer only were used as negative controls. The intra-assay variations (%CV) were 5.2%. A normal serum sample and a spiked serum sample were used as internal controls, with inter-assay CV of 21% and 9%, respectively. No freeze/thaw interference was observed, and addition of PD-L1 or human serum albumin did not influence the results.

Soluble PD-L1 was measured using an enzyme-linked immunosorbent assay (ELISA) (PDCD1LG1, Cloud-Clone Corp., Houston, TX, USA) according to the manufacturer’s instructions. The detection range was 0.156–10 ng/mL and the minimum detectable dose was 0.056 ng/mL; samples below this cutoff were assigned this value. All samples were diluted 1:3 in PBS and analyzed in duplicate. A normal serum sample and a spike serum sample were used as internal controls.

### 4.3. PD-1 and PD-L1 Gene Expression in the Tumor Tissue, Multiplex Immunohistochemistry, and Blood Lymphocyte Counts

*PD-1* and *PD-L1* encoding mRNA levels were measured from tumor samples using digital gene expression analysis with NanoString nCounter (Nanostring Technologies, Seattle, WA, USA), as described previously [15]. Proportions of PD-1-, PD-L1-, CD3-, CD4-, CD8-, and CD68-positive cells in tumor tissues analyzed by multiplex immunohistochemistry staining were described previously [15,42]. Blood lymphocyte values were obtained from routine automated complete blood count determination from peripheral blood samples collected prior to treatment initiation.

### 4.4. Statistical Analyses

Statistical analyses were performed with IBM SPSS Statistics v.25.0 (IBM, Armonk, NY, USA). The significance of difference in sPD-1 and sPD-L1 levels between time points was evaluated with Wilcoxon signed ranks test. The difference in sPD-1 and sPD-L1 levels between groups was evaluated using the Mann–Whitney U test. Correlation analyses were performed with Spearman rank analysis. ROC curve analysis was used to determine the optimal cutoff-point for the pretreatment sPD-1 level. In the ROC curve analysis, survival outcomes were dichotomized into progression-free survival (PFS) events (relapse or death versus no relapse or death). The Kaplan–Meier method was used to estimate the survival rates and the log-rank test was used to compare the differences between the groups. The prognostic impact was estimated by Cox univariate regression analysis (95% confidence interval) with categorized values. The chi-square test and the Fisher–Freeman–Halton test were used to evaluate the differences in the frequency of prognostic factors in the patient groups. Overall survival (OS) was defined from the date of trial entry until the last follow-up or death, and PFS from the date of trial entry until relapse or death. Both OS and PFS were reported in months. *p*-values below 0.05 were considered statistically significant and all statistical tests were two-tailed.

## 5. Conclusions

Earlier studies showed that pretreatment sPD-L1 levels correlate with poor outcome in DLBCL patients treated with R-CHOP immunochemotherapy. However, adverse prognostic impact of sPD-L1 levels was not observed in patients treated with high-dose chemotherapy and autologous stem cell transplantation [31]. In this study, we demonstrated that sPD-L1 and sPD1 levels decreased in response to dose-dense immunochemotherapy in patients with high-risk DLBCL. Furthermore, high pretreatment sPD-1 levels correlated with the quantities of tumor-infiltrating PD1+ T cells, translating to poor survival, whereas no correlation was observed between sPD-L1 levels and outcome. The findings provide interesting prospects for future clinical trials in DLBCL.

## Figures and Tables

**Table 1:** Characteristics of all patients in the study and according to pretreatment sPD-1 and sPD-L1 levels.

Characteristic	All Patients, *n* (%)	Low Pretreatment sPD-1, *n* (%)	High Pretreatment sPD-1, *n* (%)	*p* Value	Low Pretreatment sPD-L1, *n* (%)	High Pretreatment sPD-L1, *n* (%)	*p* Value
Total	121 (100)	80 (66)	41 (34)		40 (50)	40 (50)	
Median age (range)	56 (21–65)	54 (21–65)	57 (30–65)		54 (21–65)	54 (22–65)	
Age							
<60 years	82 (68)	54 (68)	28 (68)	1.000	30 (75)	29 (73)	0.799
60–65 years	39 (32)	26 (32)	13 (32)		10 (25)	11 (27)	
Gender							
Male	75 (62)	46 (58)	29 (71)	0.172	25 (63)	26 (65)	0.816
Female	46 (38)	34 (42)	12 (29)		15 (37)	14 (35)	
Entity							
DLBCL NOS							
GCB	53 (44)	41 (51)	12 (29)	0.055 ^1^	18 (45)	17 (42)	0.788 ^1^
Non-GCB	36 (30)	21 (26)	15 (37)		11 (28)	12 (30)	
ND	16 (13)	10 (13)	6 (15)		2 (5)	5 (13)	
Other	16 (13)	8 (10)	8 (19)		9 (22)	6 (15)	
ECOG PS							
0–1	86 (71)	60 (75)	26 (63)	0.208	32 (80)	20 (50)	**0.005**
2–3	35 (29)	20 (25)	15 (37)		8 (20)	20 (50)	
Stage							
1–2	8 (7)	8 (10)	0 (0)	**0.050**	5 (13)	1 (3)	0.090
3–4	113 (93)	72 (90)	41 (100)		35 (87)	39 (98)	
aaIPI score							
0	4 (3)	4 (5)	0 (0)	0.341	4 (10)	0 (0)	**0.016**
1	6 (5)	4 (5)	2 (5)		2 (5)	2 (5)	
2	74 (61)	51 (64)	23 (56)		25 (63)	18 (45)	
3	37 (31)	21 (26)	16 (39)		9 (22)	20 (50)	

^1^ Comparing GCB and non-GCB. NOS, not otherwise specified; GCB, germinal center B-cell-like; non-GCB, nongerminal center B-cell-like; ND, not determined; ECOG PS, Eastern Cooperative Oncology Group performance status; aaIPI, age-adjusted International Prognostic Index. The differences in the frequency of prognostic factors in the patient subgroups were compared with chi-square test and the Fisher–Freeman–Halton tests. Statistically significant *p*-values are bolded.

**Table 2:** Multivariate Cox regression analysis.

Variable	OS			PFS		
	HR	95% CI	*p* Value	HR	95% CI	*p* Value
sPD-1, low vs. high	4.06	1.00–16.50	0.050	4.10	1.21–13.89	**0.024**
Age	1.03	0.95–1.12	0.446	1.06	0.98–1.14	0.165
ECOG PS (0, 1, 2, 3)	1.59	0.77–3.25	0.209	1.30	0.70–2.40	0.403
Stage (1, 2, 3, 4)	0.81	0.27–2.40	0.700	1.02	0.35–2.93	0.975
LDH, low vs. high	1.27	0.14–11.51	0.830	0.99	0.12–8.43	0.995
Subtype, GCB vs. non-GCB	1.27	0.37–4.34	0.706	1.07	0.36–3.17	0.899

OS, overall survival; PFS, progression-free survival; HR, hazard ratio; CI, confidence interval; sPD-1, soluble programmed cell death 1; ECOG PS, Eastern Cooperative Oncology Group performance status; LDH, lactate dehydrogenase; GCB, germinal center B-cell-like; non-GCB, nongerminal center B-cell-like. Statistically significant *p*-values are bolded. sPD-1 level, LDH value and molecular subtype were analyzed as categorized variables and the others as continuous variables.

## Data Availability

The data presented in this study are available on request from the corresponding author. The data are not publicly available due to privacy or ethical restriction.
